# Editorial: Perspectives on new fast-acting antidepressants

**DOI:** 10.3389/fpsyt.2024.1430990

**Published:** 2024-05-27

**Authors:** Rodrigo Simonini Delfino, Balwinder Singh, Acioly L. T. Lacerda

**Affiliations:** ^1^ Department of Psychiatry, Federal University of São Paulo, São Paulo, Brazil; ^2^ Department of Psychiatry and Psychology, Mayo Clinic, Rochester, MN, United States

**Keywords:** depression, antidepressants, rapid acting antidepressants, ketamine, NMDA antagonist

Classic antidepressants typically require weeks to months to produce clinically significant effects. Considering that only one-third of patients achieve remission from depression with their first prescribed drug (1), a large proportion of patients will be submitted to several lengthy trials of antidepressants, and a big part of treatment time will be spent waiting and hoping for medications to end up working. Considering also that depressed individuals who commit suicide frequently seek help from mental health professionals weeks prior to completion of suicide, there is an emerging need for treatments with a faster onset of antidepressant action.

In recent years, there has been a notable emergence of compounds identified as "rapid-acting antidepressants (RAADs)", sparking significant interest in understanding the mechanisms behind these drugs’ ability to elicit such quick responses. NMDA (N-methyl-D-aspartate) antagonism, a property shared by ketamine, esketamine, and dextromethorphan, is thought to be one of the mechanisms contributing to rapid antidepressant effects. However, it’s not likely to be the ultimate pathway; instead, it acts as a mediator for the rapid release of BDNF (Brain-Derived Neurotrophic Factor).Other substances with demonstrated RAAD proprieties are scopolamine, brexanolone, zuranolone and psilocybin. Scientific papers examining the perspectives of RAADs in psychiatry have been gathered in this Research Topic.

Aiming to broaden the clinical uses of NMDA-antagonists in psychiatry, Gan et al. evaluated a new potential esketamine property: its effect on reducing postoperative depressive symptoms on patients undergoing lung cancer surgery. Examining 156 patients, in a randomized double-blind, placebo-controlled design, the authors evaluated whether intravenous intraoperative and postoperative (up to 48 hours) esketamine could reduce the development of depressive symptoms. The incidence of depressive symptoms measured by the Beck Depression Inventory-II 1 month postoperatively – was lower in the esketamine group (1.3%) as compared to the placebo (saline) group (11.8%). Two risk factors for postoperative depressive symptoms identified were preoperative anxiety and hypertension.

Yavorsky et al. stated recommendations on how to select and adapt currently used clinical rating scales for the use on clinical trials for RAADs. This is a subject of great importance, since the instruments most frequently used today were developed many decades ago, and were designed to best capture the effects of monoaminergic antidepressants. For example, scale’s items such as changes in weight and sleep may not have adequate sensitivity to evaluate efficacy in RAADs trials. Some modifications of well established clinical scales, such as the HAMD-6, may be better suited for this purpose than their original form. However, new clinical scales specifically designed to assess RAADs efficacy hold the biggest promise in the field, with the Symptoms of Major Depressive Disorder Scale (SMDDS) and the McIntyre And Rosenblat Rapid Response Scale (MARRRS) being the major representatives of this new class of instruments.

Although ketamine is most widely studied via intravenous infusions, and esketamine through intranasal administration, less is known of their profile trough subcutaneous injections. Studies have shown that the subcutaneous route for ketamine administration is a viable, low complexity procedure, and possibly associated with fewer side effects. However, the subcutaneous route has not been extensively studied in regard of its putative antisuicidal effects. This is a very important topic, since ketamine’s antisuicidal profile, although demonstrated in various clinical trials, is not universally consistent throughout the literature, and have previously yielded mixed results, sometimes attributed to the route of administration, the enantiomer used, or the chronicity of the suicidal ideation (2). Anzolin et al. outline a protocol for a prospective multicenter study examining the efficacy of subcutaneous ketamine in reducing suicidal ideation and behavior in patients with unipolar or bipolar depression. The protocol consists of 8 treatments administered twice a week, followed by 8 more, once a week, in case of adequate response to the first cycle. Follow-up evaluations will be made with 3 and 6 months of completion of the infusions. The primary outcome will be the reduction in the C-SSRS, but the study will also look at possible treatment impacts on metabolic and inflammatory markers.

The large effect sizes seen with ketamine treatment for depression and its rapid onset of action provide grounds for the study of its actions on other correlate clinical situations. One of these scenarios is catatonia. Catatonia is a severe neuropsychiatric syndrome, and a possible manifestation of severe depressive states (as well of other psychiatric disorders). Although electroconvulsive therapy (ECT) is the gold standard for treatment of catatonia, ECT is not widely available in various treatment centers, and not every patient is deemed eligible for ECT treatment. In light of these limitations, Sarma et al. presented two cases of patients with catatonia secondary to severe bipolar depression, who were referred to ECT treatment, but were deemed unfit for the treatment due to clinical comorbidities. Both patients were referred to ketamine infusions, and after the first two infusions they already presented remarkable improvement of their symptoms, and went on to show full resolution of their symptoms at the end of the treatment, returning to baseline level of functioning. The authors conclude their report encouraging further investigation of ketamine as a possible treatment for bipolar catatonia.

In summary, we have gathered relevant studies that point to the future of RAADs in psychiatry. The burgeoning field of RAADs heralds a new era in the treatment of depression, one that prioritizes quick therapeutic onset to mitigate the extensive delays and high progression rates to treatment-resistant states associated with traditional antidepressants. The evolution of the RAADs field represents a shift towards more efficient mental health interventions, promising a brighter future for patients grappling with depressive disorders.

## Author contributions

RD: Conceptualization, Supervision, Writing – original draft, Writing – review & editing. BS: Writing – original draft, Writing – review & editing. AT: Writing – original draft, Writing – review & editing.
